# Physical Performance, Sarcopenia and Malnutrition—Basic Test Set for Everyday Use in Cancer Therapy

**DOI:** 10.1002/cam4.71505

**Published:** 2026-01-04

**Authors:** S. Einhell, M. Albrecht, S. Windschüttl, A. M. Sedlmeier, F. Lüke, D. Sparrer, S. Gärditz, L. Valentini, M. Koller, T. Pukrop

**Affiliations:** ^1^ Department of Internal Medicine III, Hematology and Oncology University Hospital Regensburg Regensburg Germany; ^2^ Center for Translational Oncology University Hospital Regensburg Regensburg Germany; ^3^ Bavarian Cancer Research Center (BZKF) Regensburg Germany; ^4^ University of Applied Sciences Neubrandenburg Neubrandenburg Germany; ^5^ Center for Clinical Studies University Hospital Regensburg Regensburg Germany

**Keywords:** bioelectrical impedance analysis, cancer, cancer patients, fitness, outpatient clinic, physical performance

## Abstract

**Purpose:**

Testing physical performance, malnutrition, and sarcopenia is recommended in numerous guidelines for hematological‐oncological patients. However, due to economic conditions and the large number of test options, these tests have not made it into clinical routine. The aim of this study is to propose a practical compromise that could allow routine testing under the current conditions.

**Methods:**

We conducted a prospective, monocentric, small‐scale cohort study. In our interdisciplinary tumor outpatient clinic, we investigated time required for malnutrition screening (PG‐SGA long form), malnutrition diagnostics (GLIM), and the algorithm for sarcopenia diagnostics (EWGSOP II) in a cohort of 29 cancer patients. Further, correlation analyses of the individual tests of the examination instruments were used to achieve a possible reduction in the required number of test methods.

**Results:**

In this cohort, we identified a substantial number of patients with malnutrition (55.2%) and risk of sarcopenia (20.7%). However, this still requires 19 min even at the third visit of the patients. In order to identify potential duplications and thus unnecessary time expenditure, the tests were correlated with each other. This revealed that the assisted tests could be reduced to 3, which took only 6 min in total during the third visit.

**Conclusion:**

Within this study, we were able to reduce the number of tests to three—grip strength, bioelectrical impedance analysis, and Sit‐to‐Stand—without failing to diagnose 96.6% of the patients with risk of malnutrition and sarcopenia. This allowed us to reduce the necessary assistance time by 70% and thus made it usable in the daily routine.

## Graphical abstract

“3 in 6 Physical Performance Test”—Basic test set for everyday use in cancer therapy. Our “3 in 6 Physical Performance Test” (BIA, Hand grip strength, Sit‐to‐Stand), which takes less than 6 min in total, can be used as a starting point for nutritional medicine, physiotherapy and to assess the patient's ability to undergo therapy.

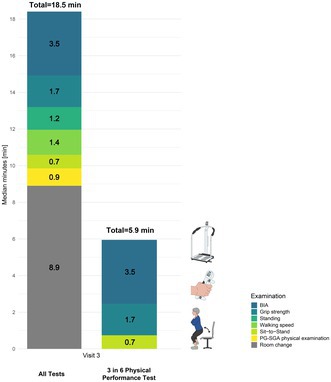

**Abbreviations**
BIA: bioelectrical impedance analysis
BMI: body mass index
ECOG: Eastern Cooperative Oncology Group
EWGSOP II: European Working Group on Sarcopenia in Older People II
GLIM: Global Leadership Initiative on Malnutrition
ICT: Interdisciplinary Center for drug‐based tumor therapy, Interdisziplinäres Zentrum für medikamentöse Tumortherapie
PG‐SGA (lf): Patient‐Generated Subjective Global Assessment (long form)
PhA: phase angle
QoL: quality of life
SARC‐F: Strength, Assistance with walking, Rise from a chair, Climb stairs and Falls
SPPB: Short Physical Performance Battery
UKR: University Hospital Regensburg, Universitätsklinikum Regensburg

## 1 Introduction

For cancer patients, tumor‐specific features such as histological and molecular tumor type, stage, and pretreatments are the main factors determining the treatment algorithms in the current guidelines. Despite additive diagnostics and determination of detailed laboratory parameters and genetic tests, the best treatment strategy addressing tumor therapy and quality of life (QoL) remains difficult, particularly in critical cases and higher therapy lines [1, 2, 3]. In order to find the best treatment option, it is desirable to have a standardized, objective, easy to collect, patient‐specific but cross‐entity score.

It has long been known that malnutrition and sarcopenia—loss of muscle mass, strength, and function—play a major role in cancer patients and have a significant impact on survival, QoL, and adherence to therapy [4, 5, 6, 7, 8, 9]. For this reason, both the S3 guideline “Klinische Ernährung in der Onkologie (Clinical Nutrition in Oncology)” and the European Society for Clinical Nutrition and Metabolism Guidelines recommend regular screening [5]. Since 2019, the criteria of the Global Leadership Initiative on Malnutrition (GLIM) have been the international standard for malnutrition diagnostics [10]. EWGSOP II (European Working Group on Sarcopenia in Older People) also developed an algorithm for the diagnosis of sarcopenia in older people, which is also used for cancer patients [11]. These diagnoses form the basis of a nutrition and exercise intervention, which in turn has a positive effect on survival and QoL [4, 12, 13].

However, these parameters are rarely used in routine clinical practice [14]. For this reason, we decided to carry out a feasibility study on malnutrition and sarcopenia diagnostics in an outpatient setting as part of an interdisciplinary cancer outpatient clinic (ICT, Interdisziplinäres Zentrum für medikamentöse Tumortherapie) at the University Hospital Regensburg (UKR). Our aim is to record the essential clinical information for optimal patient treatment and to reduce the necessary examinations to a minimum number (basic test set) in order to save resources and make it feasible for daily routine. This manuscript reports on this pilot study.

## 2 Material & Methods

### 2.1 Setting

Established in 2016, the ICT is a university‐based, interdisciplinary, outpatient clinic at the University Hospital Regensburg (UKR). Around 60–80 adult patients with various tumor diseases who require systemic therapy (including supportive measures) are treated at the ICT on a daily basis by medical doctors (MD) from seven different disciplines.

In May 2018, the concept of quality of life guides was introduced, and the initial one staff position has since been expanded. These employees serve as primary contact points for patients and their relatives in the event of problems, provide assistance with organizational issues, and arrange contacts with other specialist areas such as social services, psycho‐oncology, or the nutrition team.

### 2.2 Study Design

This is a prospective, monocentric, small‐scale cohort study. The ethics committee of the University of Regensburg has issued an ethics vote for the entire project (reference 21–2471‐101).

Patients of legal age with tumor diseases who were at the beginning of their systemic tumor therapy or before a clinically indicated change in therapy were included after medical information and written consent. Patients with an Eastern Cooperative Oncology Group (ECOG) > 2, a pacemaker/defibrillator, cognitive impairment, the inability to follow the protocol, or patients already included in another study that prohibited biomaterial asservation were excluded.

### 2.3 Trail Workflow

The trail workflow was developed by an interdisciplinary team (MDs, nurses, a registered dietitian, the Center for Clinical Studies) in order to test well‐established questionnaires and measurements from malnutrition and sarcopenia diagnostics in the daily routine of the oncological outpatient clinic. Patients were considered at the start or change of cancer‐directed therapy. Visits were carried out at intervals of 3–4 weeks, depending on the therapy applied. As described above, the study consisted of two parts: (1) standardized questionnaires and (2) active measurements.

#### 2.3.1 Standardized Questionnaires

The following questionnaires were included: (i) the PG‐SGA short form as part of the ***P**atient‐**G**enerated **S**ubjective **G**lobal **A**ssessment **l**ong **f**orm* (PG‐SGA lf) for malnutrition, and (ii) the ***S**trength, **A**ssistance with walking, **R**ise from a chair, **C**limb stairs and **F**alls* (SARC‐F) for sarcopenia screening. Both were in German, whereby questions of the PG‐SGA were modified for better understanding without changing the meaning [15, 16]. These questionnaires were completed independently by the patient during the waiting period and therefore were not included in the timekeeping.

The sarcopenia screening according to SARC‐F assesses the patient's mobility in everyday life as well as the risk of falling and indicates sarcopenia at ≥ 4 points [11]. The malnutrition assessment according to PG‐SGA lf consists, besides the questionnaire (PG‐SGA short form), of a specific and additional clinical examination (PG‐SGA physical examination) which was performed mainly by dietitian or MD. The evaluation by the categories “no malnutrition”, “moderate/suspected malnutrition” and “severe malnutrition” was carried out according to the established protocols [17, 18].

The malnutrition diagnosis according to GLIM by the categories “no malnutrition”, “moderate” and “severe” was carried out according to the established protocols [10].

#### 2.3.2 Active Measurements

In this study, all measurements were carried out with assistance by a dietitian and regardless of the screening results. The time required for these tests was analyzed using an Android application “Multi Timer Stop Watch” during the three visits (V) (V1 *n* = 29, V2 *n* = 25, V3 *n* = 20). The time measurements also included instruction of the patients and the total time, including room changes. We performed the ***b**ioelectrical **i**mpedance **a**nalysis* (BIA, mBCA 515 from seca, Hamburg, Germany), the grip strength (MAP 80K1S dynamometer from Kern, Balingen‐Frommern, Germany), and the ***S**hort **P**hysical **P**erformance **B**attery* (SPPB) [10, 11].

### 2.4 Statistical Analysis

The statistical analysis was based on descriptive methods and correlation coefficients. We calculated absolute and relative frequencies for categorical variables and median values and interquartile ranges for continuous measures.

Correlation analyses were carried out to record the statistical relationships between the variables. Potential correlations should help to form hypotheses, which are validated in further studies. Spearman correlation coefficients were computed within the tests carried out for malnutrition and sarcopenia screening (PG‐SGA lf, GLIM, PhA; SARC‐F, Grip Strength, SPPB, PhA) and between the individual tests of the SPPB. Spearman's rank correlation was applied specifically due to the ordinal nature of certain variables and the potential non‐linearity of associations [19]. This approach allowed us to reduce redundancy between screening instruments and optimize the selection of relevant assessments, ultimately minimizing the number of tests required and reducing the overall burden of testing on participants.

For studies with small sample sizes, it is generally advisable not to rely on *p*‐values and confidence intervals in the statistical analysis. In this setting, *p*‐values can be misleading as they are heavily influenced by sample size and variability. This can lead to incorrect conclusions when interpreting the significance of results [20].

All data analyses were performed using R 4.3.1 and SPSS (29.0.0.0).

## 3 Results

In the study period from April to August 2022, 29 cancer patients with the characteristics shown in Table 1 were included in this prospective trial. Despite the slightly increased median BMI (25.8 kg/m^2^), the initial screening for malnutrition according to PG‐SGA lf showed severe malnutrition in 6.9% (*n* = 2), a moderate malnutrition or suspected malnutrition in 48.3% (*n* = 14) (Data S1A). The malnutrition diagnosis according to GLIM subsequently revealed severe malnutrition in 24.1% (*n* = 7) and moderate malnutrition in 3.4% (*n* = 1) (Data S1B). Overall, 6 of 29 patients (20.7%) were at risk of sarcopenia according to SARC‐F (≥ 4 points) (Data S1C).

**TABLE 1:** Patient characteristics: Sex, age, height, weight, body mass index, and Eastern Cooperative Oncology Group (ECOG), as well as diseases.

	*n* (%) or median (Q1–Q3)
Sex	
Men	20 (69.0)
Women	9 (31.0)
Age [years]	58.0 (56.0–68.0)
Height [cm]	173.1 (165.7–180.0)
Weight [kg]	78.9 (64.5–88.3)
Body mass 11index [kg/m^2^]	25.8 (22.8–27.9)
ECOG	
1	26 (89.7)
2	3 (10.3)
Diseases	
Multiple myeloma	10 (34.5)
Non‐Hodgkin‐Lymphoma	9 (31.0)
Mamma carcinoma	2 (6.9)
Acute myeloid leukemia	1 (3.4)
Morbus Hodgkin	1 (3.4)
Malignant melanoma	1 (3.4)
Colon carcinoma	1 (3.4)
Oropharyngeal carcinoma	1 (3.4)
Pancreatic carcinoma	1 (3.4)
Prostate carcinoma	1 (3.4)
Others	1(3.4)

**FIGURE 1:**
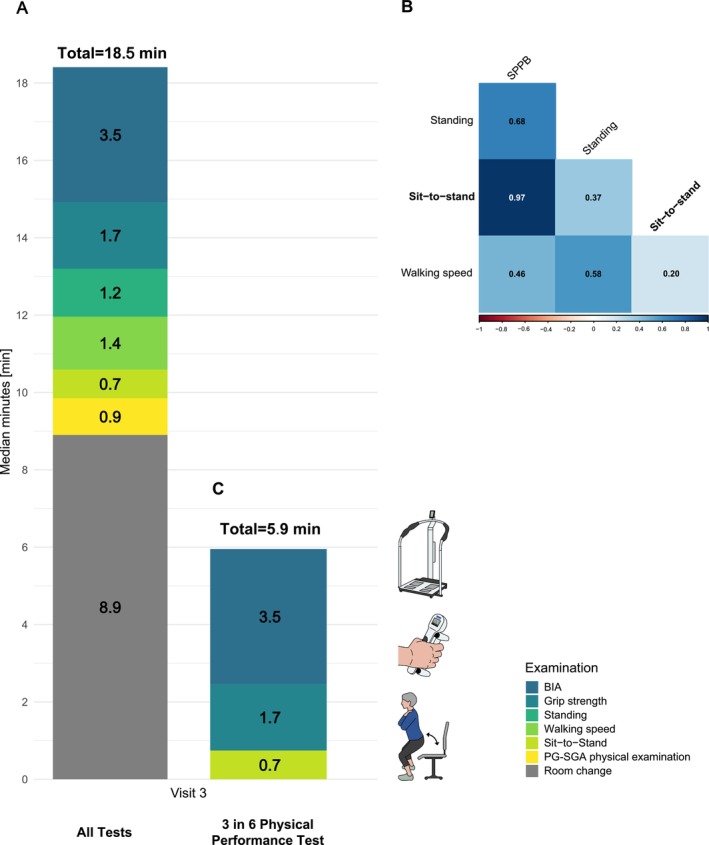
(A, C) Total median duration of all examinations in comparison to the “3 in 6 Physical Performance Test”: BIA, Sit‐to‐Stand and grip strength at visit 3. The chosen examinations last less than 6 min whereas all investigations take almost 19 min. (B) Correlations during the initial visit: Correlation of the total score and the 3 individual components of the SPPB: Sit‐to‐Stand (STS), Standing and 4 m Walking speed. BIA: bioelectrical impedance analysis; SPPB: Short Shysical Serformance Battery.

To assess feasibility under the current economic conditions, time for individual tests and all examinations, including room changes, was measured over three visits. The first visit resulted in a median total time of approx. 33 min, which was reduced to approx. 19 min over the course of the three visits (data not shown). The room changes required for the Walking speed test and PG‐SGA physical examination were particularly time‐consuming (Visit 3: 8.9 min, Figure 1A).

Since the required time of approx. 19–33 min is not feasible in everyday clinical practice, our goal was to reduce the number of measurements without losing conspicuous patients.

Concerning the individual components of the SPPB which all check the lower extremities and balance, correlation analysis between the Sit‐to‐Stand test and the overall result shows a high correlation (*r*_s_ = 0.97) (Figure 1B) and therefore the Sit‐to‐Stand is the central test of the SPPB. In addition, leaving out the Standing and especially Walking speed test helps to avoid time‐consuming changes of room. Further, SPPB itself only shows a low positive correlation to grip strength (*r*_s_ = 0.17) and PhA (*r*_s_ = 0.25) (data not shown). Because of these low correlations, there is no strong indication to the device measurements.

The grip strength itself, which addresses the upper extremities, only shows a clear correlation with the phase angle (PhA) (*r*_s_ = 0.54, data not shown).

**FIGURE 2:**
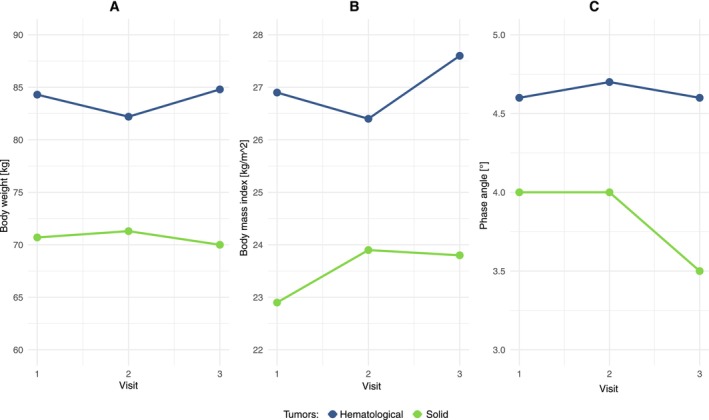
Median body weight, body mass index (BMI), phase angle (PhA) over the course of 3 visits. In contrast to the course of body weight and BMI, which show no changes, there is a clear drop in the PhA for patients with solid tumors.

The PhA is an important parameter in the BIA measurement. With no change in median weight or BMI over the course of 3 visits, a significant drop in the PhA was observed in patients with solid tumors; patients with hematologic neoplasms showed a constant course (Figure 2A–C). One case of a patient with primary metastatic pancreatic carcinoma undergoing third‐line therapy is particularly impressive, with a significantly reduced PhA of 2.8° at the beginning of the third line (reference values between approx. 5°–7° depending on age). This decreased to 2.2° over the course of the following month, and the patient died approximately 1 month after this measurement (data not shown).

The screening questionnaires and assessments give no clear indication of the device measurements (PhA from BIA or grip strength). The correlation analysis shows a high positive correlation between PG‐SGA lf and GLIM (*r*_s_ = 0.62, data not shown) and each a low negative or no correlation to PhA (PG‐SGA lf and PhA *r*_s_ = −0.23; GLIM and PhA *r*_s_ = −0.07, data not shown). SARC‐F shows a clearly negative correlation with SPPB (*r*_s_ = −0.44) and a low negative correlation with grip strength (*r*_s_ = −0.16) and PhA (*r*_s_ = −0.12) (data not shown).

Taking into account the measurements and correlation analyses carried out, the avoidance of room changes due to the time required, and the wish to address upper and lower extremities, the Sit‐to‐Stand from the SPPB, the BIA measurement with the PhA and the grip strength prove to be important and practicable clinical tests. The Sit‐to‐Stand and grip strength measurement address the lower and upper extremities. In addition to the PhA, the BIA provides numerous other parameters. The screening questionnaires and the PG‐SGA physical examination showed no additional benefit compared to the measurements performed. Moreover, the additional PG‐SGA physical examination was time‐consuming with about 1 min per patient at visit 3 plus a room change.

The pilot study indicates that the number of performed tests and questionnaires could be significantly reduced to the basic test set—Sit‐to‐Stand, BIA and measurement of grip strength—while still covering over 96.6% (28/29) of the patients with pathological results (Table S2). Finally, the chosen basic test set took only 5.9 min (Figure 1C).

## 4 Discussion

Within this pilot study, 55.2% of cancer patients showed signs of malnutrition using established tests (PG‐SGA lf) and 20.7% were at risk of sarcopenia (SARC‐F) before start or change of a systemic therapy. This observation is not a local peculiarity, but it has been described in numerous studies that a certain number of patients with signs of malnutrition or sarcopenia are overlooked in conventional routine without systematic screening [21, 22, 23, 24]. Thus, we confirm again the need for systematic assessment in this study.

Parameters relating to nutritional status and sarcopenia can provide important information on the physical fitness and, thus, the therapeutic capability of cancer patients [25]. They also enable early initiation of nutritional or exercise therapy, which can have a positive effect on survival and QoL [4, 13].

However, human resources and time are often critical in integrating these assessments into routine care. As a result, economic considerations often impede the time‐consuming systematic implementation of a routine screening [26]. Even in our day clinic, a duration of approximately 19–33 min exceeds the available capacity. In order to satisfy both economic considerations and acceptance by staff and patients in the current situation, it is necessary to optimize the recording of the most important parameters while maintaining the quality of the information. With this in mind, this study not only carried out the tests, but also measured the time required to perform and prepare them.

Based on the correlation analyses and the time measurements which reflect the personnel costs, we would recommend the following procedure as a basic test set: the BIA, the solitary Sit‐to‐Stand and the grip strength measurement (“3 in 6 Physical Performance Test”). Concerning the SPPB, the Sit‐to‐Stand test alone shows a very high correlation with the overall SPPB value. By concentrating on this test, 2.6 test minutes could be saved and time‐consuming room changes could be avoided. In further studies, the distance to the center, which plays a role especially for the rural region of eastern Bavaria, must also be considered. In this respect, a Sit‐to‐Stand test carried out at home could provide an initial starting point, which, as an Australian study has shown, compares well with the implementation with professional assistance [27]. With the help of grip strength and the Sit‐to‐Stand test, both the lower and upper extremities are included in the assessment.

The BIA measurement itself provides numerous parameters, among which the PhA, in particular, can provide an important indication of the further clinical course [28]. A large review with 273 studies and a total of approx. 78,000 oncologic patients was able to show that BIA measurements can be used to assess preoperative risk, shorten hospital stays, and provide information on prognosis [29]. The connection between a low PhA and low quality of life has also been described several times in the literature [30].

This proposed basic test set (Sit‐to‐Stand, Grip strength, BIA) would need a median total time of 5.9 min, retain essential parameters for recording physical performance and could be established in daily routines. This could enable significantly more clinics and outpatient clinics to examine and support patients with regard to malnutrition and sarcopenia. On the one hand, this would benefit the patients themselves, including increasing treatment adherence and quality of life, and on the other hand, it could also save personnel and future treatment costs. The results of an online survey of German dietitians conducted in February 2020 show that almost half of the 1311 participants care for 100–250 inpatients each, with less than half of the facilities having a nutrition team [31]. In the USA, a survey in the outpatient sector revealed a care ratio of 1:2308 [32].

However, our current investigations were only carried out on a small number of patients as part of a pilot study, which means that the statements can only be used to a limited extent. The correlations found and thus the reduction to the 3 tests described must be investigated and confirmed in further studies with a larger number of patients. Furthermore, it is important to consider and examine the very different diseases in hemato‐oncology. For example, lymphoma patients and oncology patients such as head and neck or gastrointestinal cancer patients, which already have a higher risk of sarcopenia per se [21], represent a very different patient clientele. It also makes sense to take a closer look at the monetary aspect in further studies in order to emphasize the feasibility in everyday clinical practice.

In future, the use of the “3 in 6 Physical Performance Test” could save time for staff and patients and thus reduce the workload and costs at the centers without compromising the quality of care but potentially improving the quality of life of individual patients.

## 5 Conclusion

This analysis regarding extended malnutrition and sarcopenia diagnostics to determine patients' physical fitness in the context of an interdisciplinary hemato‐oncology outpatient clinic confirmed the need for systematic screenings and tests. Both of the above‐mentioned characteristics are relevant patient features that show abnormalities in a considerable number of cancer patients and should be taken into account in the treatment of patients.

Professional implementation requires a full‐time employee. Since this is not usually available in routine practice, but an additional gain in information regarding therapy and patient well‐being is assumed, a reduced survey using BIA, grip strength measurement and Sit‐to‐Stand test (“3 in 6 Physical Performance Test”) was identified and is to be investigated in further studies.

These 3 examinations, which take less than 6 min in total, can then be used as a starting point for nutritional medicine, physiotherapy, and to assess the patient's ability to undergo therapy.

## Author Contributions

**A. M. Sedlmeier:** data curation (equal), formal analysis (equal), visualization (equal), writing – original draft (equal), writing – review and editing (equal). **D. Sparrer:** visualization (supporting). **F. Lüke:** conceptualization (equal), formal analysis (equal), writing – original draft (supporting). **M. Albrecht:** data curation (equal), investigation (equal), methodology (equal). **M. Koller:** conceptualization (equal), writing – original draft (equal), writing – review and editing (equal). **S. Einhell:** conceptualization (equal), data curation (lead), formal analysis (equal), investigation (equal), methodology (equal), project administration (lead), validation (lead), visualization (equal), writing – original draft (lead), writing – review and editing (lead). **S. Gärditz:** formal analysis (equal), investigation (equal), writing – original draft (supporting). **S. Windschüttl:** conceptualization (equal), data curation (equal), investigation (equal), methodology (equal). **T. Pukrop:** conceptualization (equal), data curation (equal), supervision (lead), writing – original draft (equal), writing – review and editing (equal). **L. Valentini:** conceptualization (equal), writing – original draft (equal), writing – review and editing (equal).

## Funding

This project was supported by the BZKF (Bavarian Cancer Research Center). The authors declare that no other funds, grants, or other support were received during the preparation of this manuscript.

## Ethics Statement

This study was performed in line with the principles of the Declaration of Helsinki. Approval was granted by the Ethics Committee of the University of Regensburg (reference 21‐2471‐101).

## Consent

All patients consented to take part in this study.

## Conflicts of Interest

The authors declare no conflicts of interest.

## Supporting information


**Data S1:** Initial screening according to PG‐SGA long form, GLIM and SARC‐F. (A) Screening for malnutrition according to PG‐SGA lf during the first visit in 29 patients. Severe malnutrition was detected in 2 patients, no malnutrition in 13 patients and moderate or suspected malnutrition in 14 patients. (B) Malnutrition diagnosis according to GLIM during the first visit in 29 patients. Severe malnutrition was detected in 7 patients, moderate malnutrition in 1 and no malnutrition in 21 patients. (C) Screening for sarcopenia according to SARC‐F. Sarcopenia was suspected in 6 out of 29 patients, as their score was ≥ 4. GLIM: Global Leadership Initiative on Malnutrition; PG‐SGA lf: Patient‐Generated Subjective Global Assessment long form; SARC‐F: Strength, Assistance with walking, Rise from a chair, Climb stairs and Falls.

**Table S2:** Test results and abnormalities visit 1.

## Data Availability

Data generated and analyzed during the current study contain sensitive clinical patient information and are therefore not publicly available due to data protection and privacy regulations. Access to the data may be granted in anonymized form upon reasonable request.
