# Supplementary figures and images for: Physical Performance, Sarcopenia and Malnutrition—Basic Test Set for Everyday Use in Cancer Therapy
Source: Cancer Med. 2026 Jan 4;15(1):e71505. doi: 10.1002/cam4.71505 (PMC12766154; doi:10.1002/cam4.71505)

**A**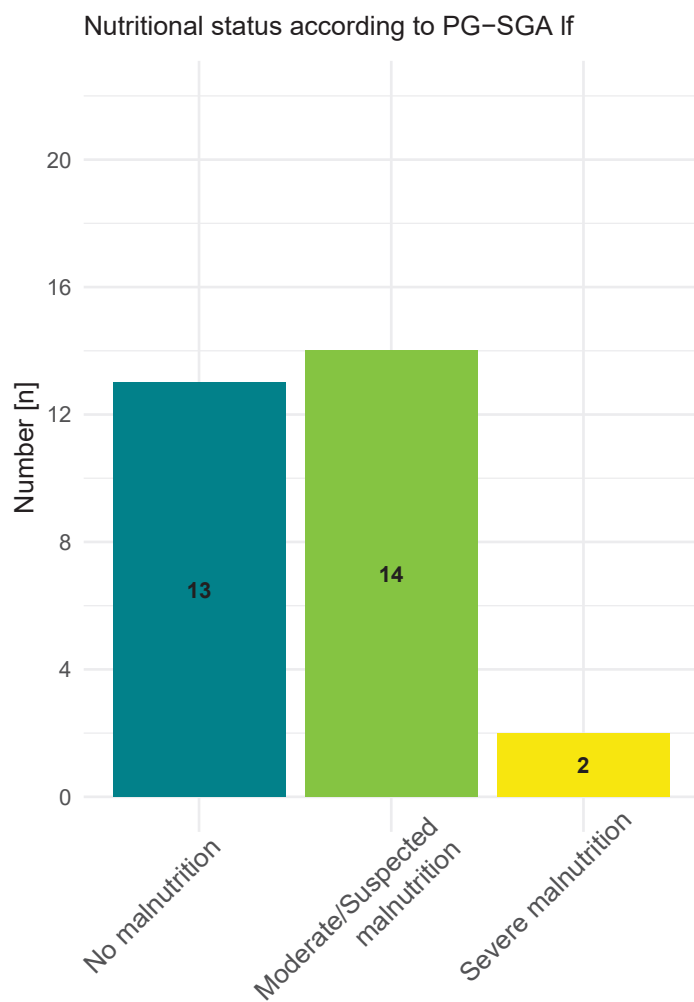**B**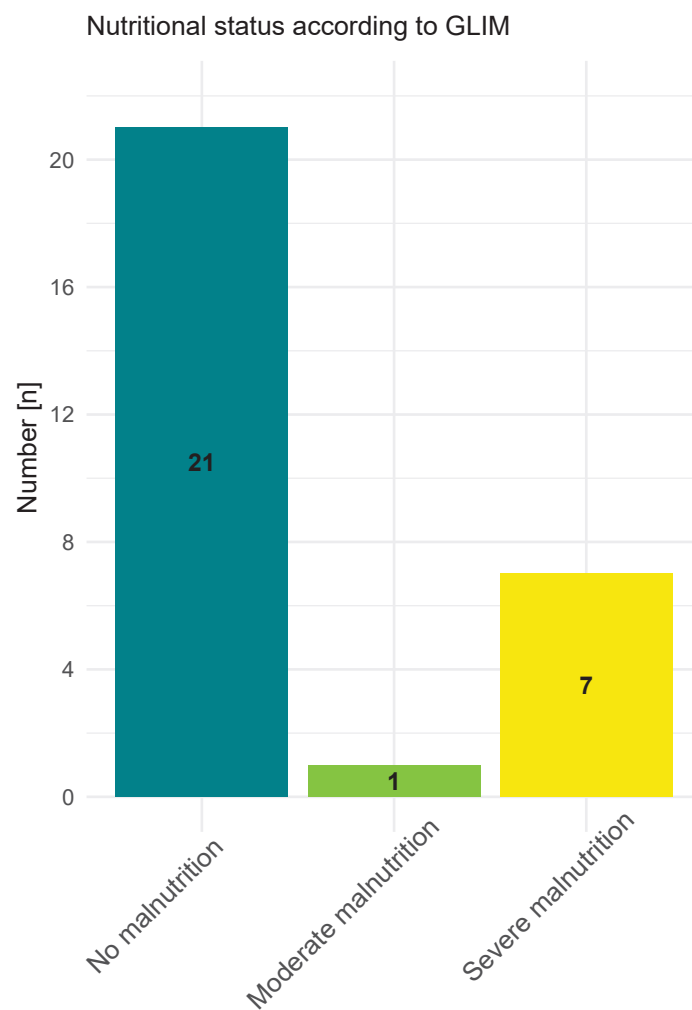**C**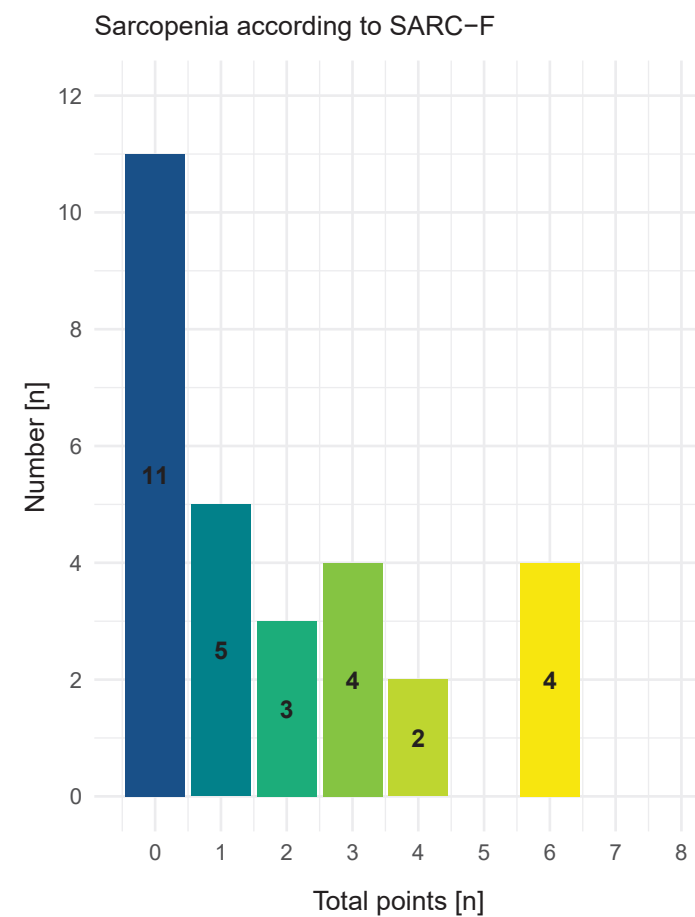

Supplement: Supplementary file 1 — Data S1: Initial screening according to PG‐SGA long form, GLIM and SARC‐F. (A) Screening for malnutrition according to PG‐SGA lf during the first visit in 29 patients. Severe malnutrition was detected in 2 patients, no malnutrition in 13 patients and moderate or suspected malnutrition in 14 patients. (B) Malnutrition diagnosis according to GLIM during the first visit in 29 patients. Severe malnutrition was detected in 7 patients, moderate malnutrition in 1 and no malnutrition in 21 patients. (C) Screening for sarcopenia according to SARC‐F. Sarcopenia was suspected in 6 out of 29 patients, as their score was ≥ 4. GLIM: Global Leadership Initiative on Malnutrition; PG‐SGA lf: Patient‐Generated Subjective Global Assessment long form; SARC‐F: Strength, Assistance with walking, Rise from a chair, Climb stairs and Falls. [file CAM4-15-e71505-s001.pdf]
