# Supplementary material for: Physical Performance, Sarcopenia and Malnutrition—Basic Test Set for Everyday Use in Cancer Therapy
Source: Cancer Med. 2026 Jan 4;15(1):e71505. doi: 10.1002/cam4.71505 (PMC12766154; doi:10.1002/cam4.71505)
Supplement: Supplementary file 2 — Table S2: Test results and abnormalities visit 1. [file CAM4-15-e71505-s002.docx]

**Supplement 2 Table 2 Test results and abnormalities visit 1:** Results of the questionnaires and examinations during the 1st visit for the 29 patients are shown. Abnormalities were marked as follows (grey): SARC-F ≥ 4 points; SPPB ≤ 8 points; STS < 15 sec; GS m < 27kg, f < 15kg; PhA <5°, PG-SGA lf 1 or 2 points; GLIM 1 or 2 points. By reducing the tests and questionnaires to BIA, GS and STS, no patient is missed in the context of sarcopenia diagnostics and only H-010 in the context of malnutrition diagnostics. ND: not done; BIA: Bioelectrical Impedance Analysis; GLIM: Global Leadership Initiative on Malnutrition; GS: Grip strength; PG-SGA lf: Patient-Generated Subjective Global Assessment long form; PhA: phase angle; SARC-F: Strength, Assistance with walking, Rise from a chair, Climb stairs and Falls; SPPB: Short Physical Performance Battery; STS: Sit-to-Stand Test.

|  | **SARC-F [pts]** | **SPPB [pts]** | **STS [sec]** | **GS [kg]** | **PhA [°]** | **PG-SGA lf [pts]** | **GLIM [pts]** |
| --- | --- | --- | --- | --- | --- | --- | --- |
| **H-001** | 6 | 9 | 23.0 | 48.0 | 5.7 | 1 | 0 |
| **H-002** | 2 | 12 | 11.0 | 50.0 | 5.4 | 1 | 0 |
| **H-003** | 0 | 10 | 16.5 | 40.9 | 6.0 | 0 | 0 |
| **H-004** | 1 | 10 | 15.1 | 30.2 | 4.2 | 0 | 0 |
| **H-005** | 6 | 9 | 24.3 | 32.2 | 4.8 | 0 | 0 |
| **H-006** | 6 | 5 | 20.0 | 54.2 | 5.0 | 1 | 2 |
| **H-007** | 0 | 9 | 21.9 | 41.4 | 4.3 | 0 | 0 |
| **H-008** | 0 | 10 | 15.3 | 43.3 | 4.2 | 0 | 0 |
| **H-009** | 0 | 9 | 17.4 | 27.9 | 4.5 | 1 | 1 |
| **H-010** | 1 | 11 | 13.5 | 46.2 | 5.5 | 1 | 2 |
| **H-011** | 2 | 7 | 16.8 | 22.0 | 2.6 | 1 | 2 |
| **H-013** | 0 | 10 | 14.4 | 51.1 | 5.9 | 0 | 0 |
| **H-014** | 6 | 7 | ND | 27.3 | 3.6 | 1 | 0 |
| **H-015** | 0 | 10 | 16.6 | 23.4 | 4.8 | 0 | 0 |
| **H-017** | 2 | 9 | 18.9 | 52.8 | 5.2 | 0 | 0 |
| **H-018** | 2 | 7 | 28.7 | 38.5 | 4.7 | 1 | 0 |
| **H-020** | 1 | 9 | 18.9 | 52.3 | 4.4 | 1 | 2 |
| **H-021** | 3 | 10 | 15.5 | 27.3 | 4.1 | 0 | 0 |
| **H-022** | 3 | 6 | ND | 29.3 | 3.2 | 1 | 0 |
| **H-023** | 4 | 9 | 17.6 | 22.8 | 4.2 | 1 | 0 |
| **H-024** | 0 | 10 | 14.6 | 26.7 | 3.9 | 0 | 0 |
| **H-025** | 0 | 10 | 15.9 | 44.8 | 4.9 | 0 | 0 |
| **S-001** | 1 | 9 | 19.5 | 51.8 | 4.3 | 0 | 0 |
| **S-002** | 0 | 11 | 12.8 | 52.0 | 4.0 | 1 | 0 |
| **S-003** | 0 | 9 | 24.4 | 20.1 | 2.8 | 1 | 0 |
| **S-004** | 4 | 10 | 14.6 | 20.6 | 4.0 | 0 | 0 |
| **S-005** | 1 | 9 | 17.6 | 37.1 | 3.6 | 2 | 2 |
| **S-006** | 3 | 10 | 13.8 | 34.0 | 4.1 | 1 | 2 |
| **I-001** | 3 | 8 | ND | 24.7 | 4.6 | 2 | 2 |
